# White Matter Fiber Tractography Using Nonuniform Rational B-Splines Curve Fitting

**DOI:** 10.1155/2018/8643871

**Published:** 2018-11-18

**Authors:** Zhanxiong Wu, Dongnan Wu, Dong Xu

**Affiliations:** Department of Electronic Information, Hangzhou Dianzi University, Hangzhou 310018, China

## Abstract

The study of neural connectivity has grown rapidly in the past decade. Revealing brain anatomical connection improves not only clinical measures but also cognition understanding. In order to achieve this goal, we have to track neural fiber pathways first. Aiming to estimate 3D fiber pathways more accurately from orientation distribution function (ODF) fields, we presented a novel tracking method based on nonuniform rational B-splines (NURBS) curve fitting. First, we constructed ODF fields from high angular resolution diffusion imaging (HARDI) datasets using diffusion orientation transform (DOT) method. Second, under the angular and length constraints, the consecutive diffusion directions were extracted along each fiber pathway starting from a seed voxel. Finally, after the coordinates of the control points and their corresponding weights were determined, NURBS curve fitting was employed to track fiber pathways. The performance of the proposal has been evaluated on the tractometer phantom and real brain datasets. Based on several measure metrics, the resulting fiber pathways show promising anatomic consistency.

## 1. Introduction

An outstanding characteristic of white matter (WM) is its fibrillar construction. WM consists of tightly packed and coherently aligned axons that are surrounded by glial cells and that often are organized in bundles [1]. Axons are protected by myelin sheaths, which restricts the free diffusion of water molecules. As a result, the micrometric movements of water molecules are hindered to a greater extent in a direction perpendicular to the axonal orientation than parallel to it. It is now generally accepted that microscopic boundaries to diffusion in WM coincide with the local orientations of WM fiber pathways [2–4]. With this feature, we can trace fiber pathways and then reveal anatomical connection between brain functional areas.

Compared to diffusion tensor imaging (DTI), high angular resolution diffusion imaging (HARDI) could resolve multiple intravoxel fiber orientations contained in a WM voxel. Moreover, HARDI just needs to sample the diffusion signal on a spherical shell as opposed to a complete three-dimensional Cartesian grid of DSI [5–7]. At present, there are numerous tracking methods based on HARDI, which could be classified into deterministic and probabilistic algorithms [8]. They exploit the diffusion anisotropy to follow fiber tracts from voxel to voxel through the brain [9]. Recently, multishell multitissue (MSMT) models have been proposed to deal with partial volume effects and can remarkably increase the precision of fiber orientations over single-shell models [10].

Streamline tracking is an important deterministic approach. Streamline tracking propagates paths within the vector field of local fiber orientations [9], providing deterministic connectivity information between different brain functional areas. Later, many variants of the streamline method have been presented. The streamline-based tracking technique is the one most commonly used in tractography, and it appears to give excellent results in many instances if the vector field is smooth and the fibers are strongly oriented along a certain direction. However, the major drawback of streamline-based methods is that the estimation error accumulates along the tracking length [11, 12]. However, the partial volume effects such as crossing, kissing, merging, and splitting in imaging voxels increase the complexity in streamline tracking.

There are also some nonstreamline tractography algorithms. In the graph-based method, each voxel is treated as a graph node, and graph arcs connect neighboring voxels. The weights assigned to each arc are the representative of both structural and diffusivity features [13]. When partial volume exists, the algorithm treats the image as a multigraph and distributes the connectivities in a weighted manner. Aranda et al. presented a particle method which was proposed to estimate fiber pathways from multiple intravoxel diffusion orientations (MIVDO) [14]. The process starts with the definition of a point in WM region in which a virtual particle is allocated. The particle is iteratively moved along the local diffusion orientations until a stopping criterion is met. The estimation of fiber pathways is determined by the particle trajectory. Galinsky and Frank proposed a method for estimating local diffusion and fiber tracts based upon the information entropy flow that computes the maximum entropy trajectories [15]. This novel approach to fiber tracking incorporates global information about multiple fiber crossings in each individual voxel. Malcolm et al. used Watson function to analyze ODF construction, which provides a compact representation of the diffusion-anisotropic signal [16]. This algorithm models the diffusion as a discrete mixture of Watson directional functions and performs tractography within a filtering framework. Recently, global tractography was proposed in [17], which aims to find the full track configuration that best explains the measured diffusion weighted imaging (DWI) data. This data-driven approach was reported that it could improve valid neural connection rate over streamline methods.

The other classes are probabilistic approaches. This class of methods utilizes a stochastic process to estimate the connection probability between brain areas. A Bayesian approach was presented in [18], and it handled noise in a theoretically justified way. The persistent angular structure (PAS) of fiber bundles was used to drive probabilistic tracts, and PDF is incorporated into the method to estimate the whole-brain probability maps of anatomical connection [19]. Using automatic relevance determination in a Bayesian estimation scheme, the tracking in a multivector field was performed with significant advantages in sensitivity [20]. The residual bootstrap method made use of spherical harmonic (SH) representation for HARDI data in order to estimate the uncertainty in multimodal q-ball reconstructions [21]. However, these methods cannot directly delineate the fiber paths in 3D brain space. Furthermore, they are very time consuming in resolving the complexity of the diffusion pattern within each HARDI voxel.

In [22, 23], the authors argued that NURBS provides a framework to characterize WM pathways. However, the determination of the parameters including control points and weights has not been discussed. This paper has comprehensively explored the tracking method based on NURBS curve fitting and has detailed how to determine the related parameters. The tracking method consists of three steps: first is the computation of ODF field from HARDI datasets; second is the selection of consecutive diffusion directions along a fiber pathway; and the last is NURBS pathway fitting. This method was evaluated on tractometer phantom and real brain datasets.

## 2. Materials and Methods

### 2.1. HARDI Datasets

Two different types of HARDI datasets are used to evaluate our approach: from the physical diffusion phantom of tractometer and from an in vivo human brain. For each dataset, we firstly constructed ODF fields using DOT method [24] and then applied the proposed algorithms to estimate fiber paths.

Phantom study was performed using data acquired from a physical diffusion phantom of tractometer. Imaging parameters for the 3 × 3 × 3 mm acquisition were as follows: field of view FOV = 19.2 cm, matrix 64 × 64, slice thickness TH = 3 mm, read bandwidth RBW = 1775 Hz/pixel, partial Fourier factor 6/8, parallel reduction factor GRAPPA = 2, repetition time TR = 5 s, and echo times TE = 102 ms. A SNR of 15.8 was measured for the baseline (*b* = 0 s/mm^2^) image. SNR of HARDI at b-values = 2000 s/mm^2^ were evaluated. The diffusion sensitization was applied along a set of 64 orientations uniformly distributed over the sphere [25]. For comparative study, the ground truth fibers are available on the website http://www.lnao.fr/spip.php?rubrique79 [25].

A healthy volunteer was scanned on a Siemens Trio 3T scanner with 12 channel coils. The acquisition parameters were as follows: two images with *b* = 0 s/mm^2^, 64 DW images with unique, and isotropically distributed orientations (*b* = 2000 s/mm^2^). TR = 6700 ms, TE = 85 ms, and voxel dimensions equal to 2 × 2 × 2 mm. The SNR is, approximately, equal to 36.

### 2.2. ODF Fields

Compared with diffusion tensor, ODFs reflect the diffusion probability along any given angular direction, and higher values indicate higher consistency between the fiber orientation and diffusion direction. ODFs can be seen as a continuous function over the sphere that encodes diffusion anisotropy of water molecules within each voxel. There are two definitions of ODF. One is Tuch's nonmarginal ODF that is defined as the radial integration of PDF and does not represent a true probability density [26, 27]. The other is marginal ODF that is introduced by Wedeen, and it is a true probability density since its integral over the sphere is one [28]. ODF peaks are assumed to correspond to the underlying fiber orientations. At present, there are several algorithms to compute ODFs from HARDI datasets. Tuch presented a simple linear matrix formulation that was provided to construct ODFs using radial basic function (RBF) [26]. Diffusion orientation transform (DOT) converts water diffusivity profiles into probability profiles under the monoexponential signal decay assumption through computing PDF at a fixed distance from the origin [24, 29, 30]. Spherical deconvolution (SD) estimates fiber orientations by assuming that a single response function can adequately describe HARDI signals measured from any fiber bundle [31]. Compared to other methods, DOT can improve the angular resolution, make the ODF sharper, and keep its accuracy and robustness to noise [27, 30]. In our work, we used DOT to construct ODFs from HARDI datasets.

After ODF fields were constructed, we detected ODF local maxima by thresholding over the sampling shell. Only those above ODF mean value would be retained. This operation can avoid the noise interference effectively [28]. Finally, ODF fields are transformed into vector fields, and we can describe a voxel using a matrix containing diffusion vectors and its corresponding diffusion probability.

(1) $$\begin{array}{c} V_{\text{voxel}}=\left[\begin{array}{c} v_{1,x}\;v_{1,y}\;v_{1,z}\;d_{1}\\ ......\\ v_{i,x}\;v_{i,y}\;v_{i,z}\;d_{i}\\ ......\\ v_{n,x}\;v_{n,y}\;v_{n,z}\;d_{n} \end{array}\right]. \end{array}$$

The term $\left[\begin{array}{ccc} v_{i,x} & v_{i,y} & v_{i,z} \end{array}\right]$ denotes a diffusion direction, and *d*_*i*_ is the diffusion probability along this orientation. In the next section, we would use this matrix to compute the control points and weights for NURBS pathway fitting.

### 2.3. Diffusion Directions along a Fiber Pathway

**Figure 1:**
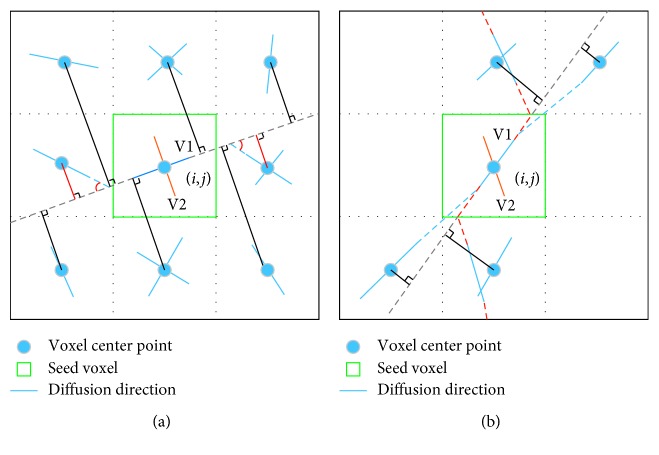
Extraction of consecutive diffusion directions along a fiber pathway. V1 (blue line in the seed voxel) and V2 (orange line in the seed voxel) denote the two diffusion directions in the seed voxel (the green square). The dark solid line denotes the distance between V1 and the center of the neighbor voxels. (a) Finding the consecutive directions under the constraints of distance, angle and length. The red lines denote the distances less than the threshold. The red arcs denote the angles between the consecutive directions. (b) Unreasonable pathway found with FACT.

Before we conduct NURBS tracking, the consecutive directions along the same pathway have to be extracted. The orientations of fiber populations within a voxel coincide with the local maxima of ODFs [28]. ODF value along a direction is the reflection of diffusion probability of all the water molecules in a voxel, so it is reasonable to assume that the diffusion directions always pass through the voxel center. The aim of this step is to find the consecutive directions among the neighbors of a seed voxel. Here, we presented a new algorithm to achieve the goal. For the sake of simplicity, we used a two-dimensional diagram as an example to illustrate the process, shown as Figure 1(a). Compared to FACT algorithm [32], it can improve the extraction accuracy of discrete consecutive directions along a pathway. As we can see from Figure 1(b), in FACT, an unreasonable path was found (marked by red dashed lines). But if the distance between V1 (blue line in the seed voxel) and the center points of its neighbor voxel is considered here, we could get a more reasonable pathway (marked by blue dashed lines in Figure 1(b)). The algorithm is summarized as Algorithm 1. The input parameters, including fiber length threshold *L*_th_, angle threshold *θ*_th_, and fractional anisotropy (FA) threshold *FA*_th_ should be determined according to actual situation.

### 2.4. NURBS Fitting

**Figure 2:**
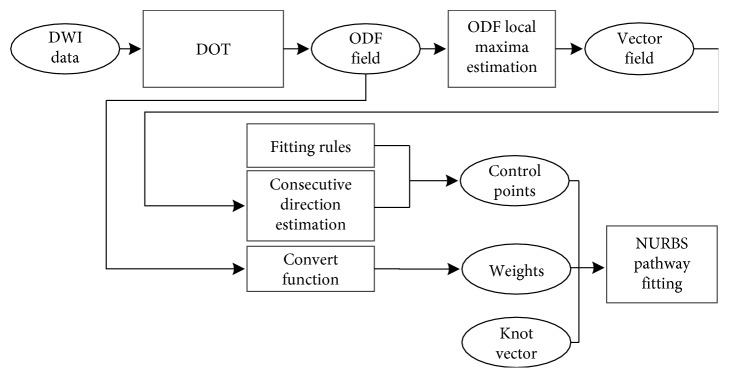
Whole process of fiber tracking based on NURBS. The knot vector was normalized, and its nodes are distributed evenly. The fitting rules are determined according to the relation between the fiber pathway and the diffusion orientation. Consecutive direction estimation is accomplished according to Algorithm 1. Convert function is as the equation given in the Algorithm 2.

NURBS is a powerful tool to describe complex curves using a small number of parameters. It is a wonderful modeling method of curves and can control the object more conveniently and efficiently than traditional modeling method [33]. The order of a NURBS curve defines the number of nearby control points that could influence any given point on the curve. In practice, cubic curves are the ones most commonly used. Higher order curves are seldom used because they may lead to internal numerical problems and require disproportionately large computation time [34–36]. The number of control points must be greater than or equal to the order of the curve. In this work, we traced nerve fiber pathways based on NURBS curve fitting. In the fitting, the parameters including control points and weights are needed. The consecutive directions were used to compute control points. The weights were computed according to *d*_*i*_. In NURBS tracking, we could use both control points and weights to hold local shape control of fiber pathways. We present two tracking methods based on NURBS according the fitting rule, including general NURBS fitting (NURBS-G) and tangent NURBS fitting (NURBS-T). The whole procedure of NURBS tracking is shown in Figure 2.

### 2.5. NURBS-T

**Figure 3:**
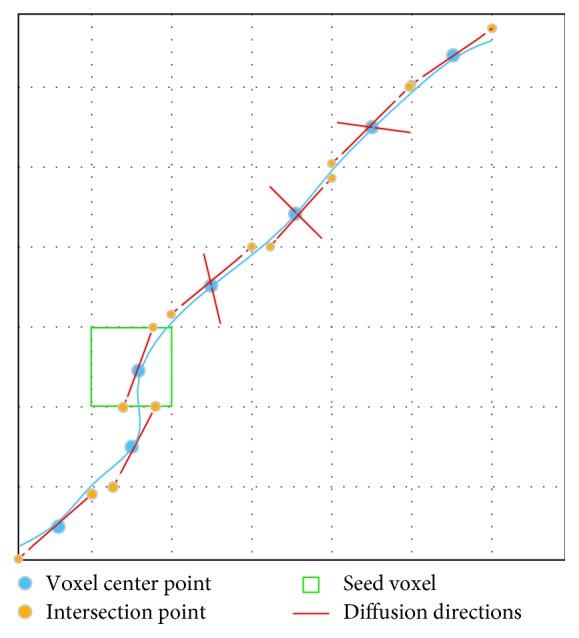
NURBS-T fiber tracking. The solid blue thick line denotes a fiber pathway. The control points consist of intersection points (yellow solid dots) and center points (blue solid dots).

A fiber pathway can be considered as a 3D curve, and its local tangent vector is consistent with the diffusion orientation [37]. According to this premise, we presented NURBS-T algorithm to trace fiber paths. To make it easier to explain, the 2D tracking process is illustrated in Figure 3. The algorithm is outlined in Algorithm 2.

### 2.6. NURBS-G

**Figure 4:**
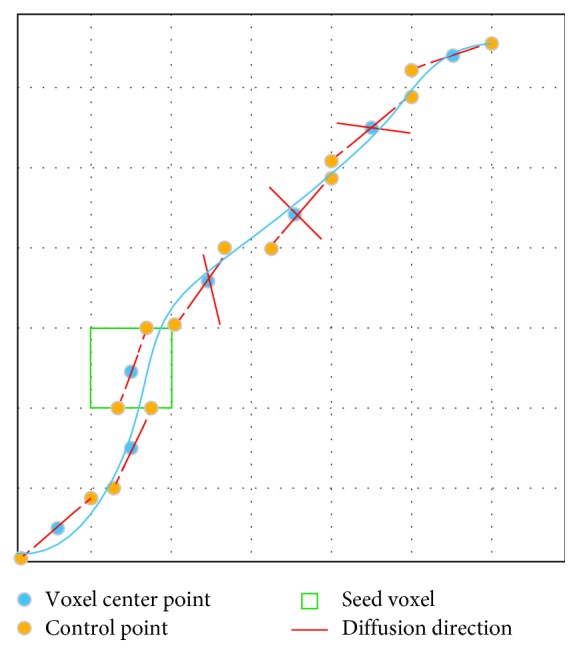
NURBS-G pathway fitting. The solid blue thick line denotes a fiber pathway. The set of control points consists of only intersection points (yellow dots).

In NURBS-G tracking, we do not consider the tangent relationship between fiber pathway and diffusion direction. The control points consist of only intersection points between the diffusion directions and the facets of the voxel. The 2D tracking process is demonstrated in Figure 4. The algorithm is outlined in Algorithm 3.

## 3. Results

**Figure 5:**
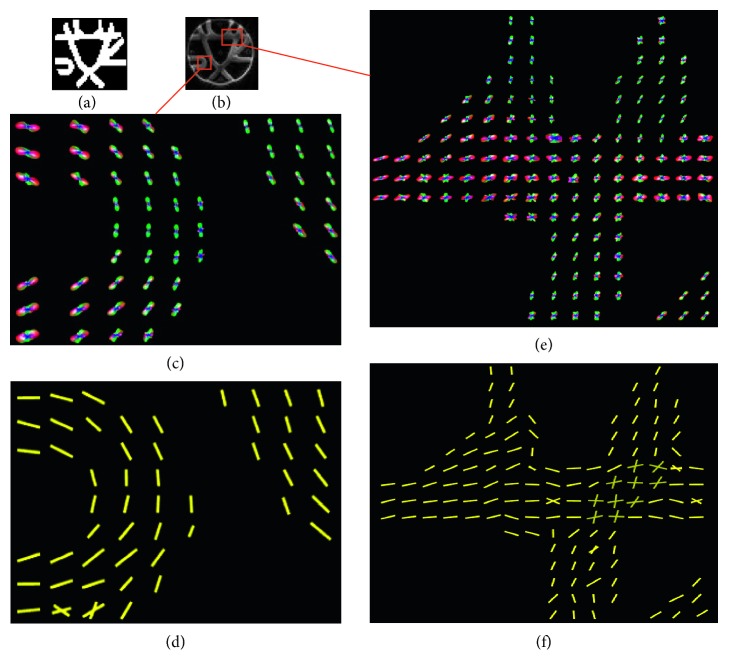
ODF and orientation fields of tractometer phantom. (a) Mask of fiber paths of the phantom, (b) T2-weighted images, (c) ODF field, (d) vector field of (c), (e) ODF field, and (f) vector field of (e).

Figure 5 shows the ODF and vector fields estimated from HARDI images of tractometer. Panel (a) is the mask of fiber pathways. We extracted the diffusion directions corresponding to ODF local maxima that are above the mean value of ODFs. Through this filtration, spurious peaks could be effectively reduced [28].

**Figure 6:**
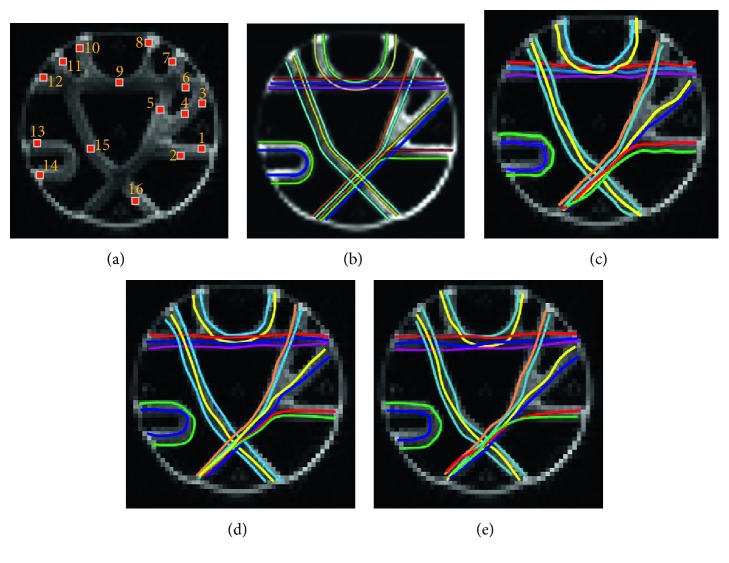
Fiber pathways tracked with FACT, NURBS-T, and NRBS-G. (a) Spatial seed points are determined according to Figure 4(a) of [25]. (b) Ground truth fiber trajectories starting from the sixteen seed points. This image is directly cited from Figure 4(c) of [25]. (c) Multidirectional streamline tracking. (d) NURBS-T tracking. (e) NURBS-G tracking.

After the vector fields were obtained, the control points and weights were computed. Next, the fiber pathways were traced with multidirectional streamline, NURBS-T, and NURBS-G. In this phantom experiment, *θ*_th_ is set to 60° and *L*_th_ is 70 mm. *FA*_th_ was not set for this test, as WM mask was provided in tractometer dataset.  Figure 6(a) shows 16 seed points selected according to [25], and 6(b) shows the ground truth fiber pathways. Figures 6(c), 6(d), and 6(e) show the tracking results.

**Figure 7:**
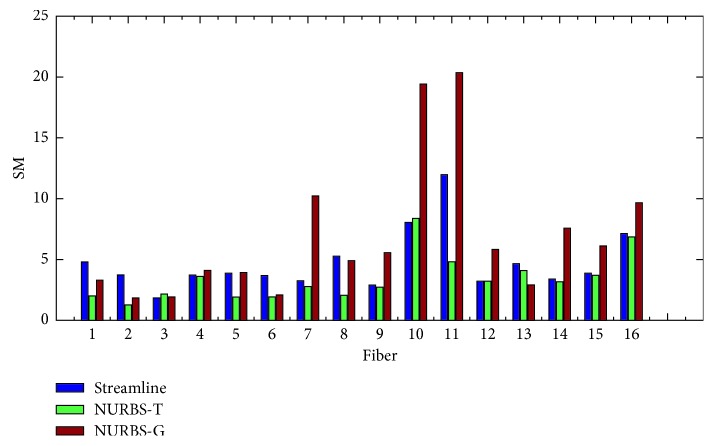
Symmetric root mean square error using the spatial metric (L2 norm).

**Figure 8:**
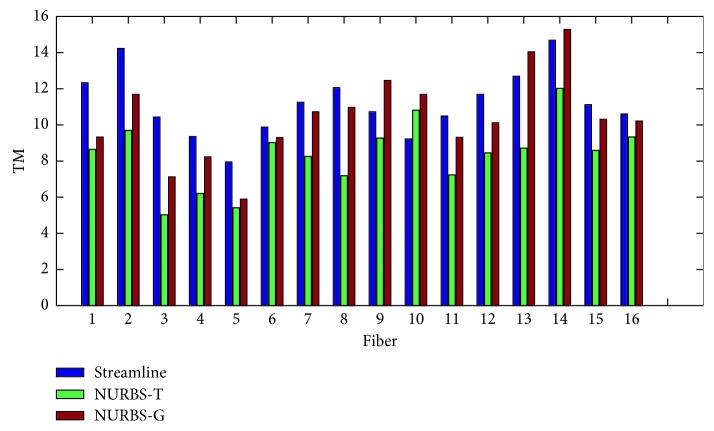
Symmetric root mean square error using the tangent metric.

**Figure 9:**
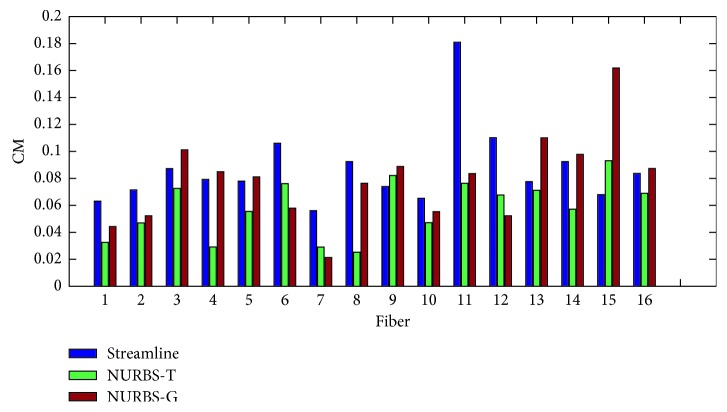
Symmetric root mean square error using the curve metric.

In order to evaluate the proposed algorithms, two kinds of measure methods were taken. One is the point-to-point performance measures; the other is the connection measures. The former includes spatial metric (SM), tangent metric (TM), and curve metric (CM) [25]. These metrics focus on the point-to-point performance from a local perspective. The latter contains valid connections (VC), invalid connections (IC), no connections (NC), valid bundles (VB), and invalid bundles (IB) [39]. From a global point of view, the connections generated by the estimated trajectories are relevant. The set of global metrics takes into account the resulting connectivity. In this experiment, we evaluated the results with both local and global metrics. Figures 7–9 show the summation of the points per metric for each method.  Table 1 shows the evaluation by using the global metrics: VC, IC, NC, VB, and IB.

We can come to that for the spatial metric NURBS-T obtains the best score except Fiber 3 and 10. For the tangent metric, NURBS-T also gets the best position except Fiber 10. For the curve metric, NURBS-T obtains the best place except for Fiber 9 and 15. Summarizing the overall performance over the three metrics, we can conclude that NURBS-T is best on the fiber pathway estimation of the phantom. For the computation time, NRBS-T recovered the previous results in about 23 minutes, and NURBS-G took about 20 minutes. The method of multidirectional streamline required 27 minutes or so to complete the task at the step of 0.02 mm. These methods were all implemented in Matlab R2014b running on the computer possessing 8G RAM and Intel Core i5-7200U.

From the above analysis, NURBS-T presents competitive results for both kinds of measure metrics. Furthermore, we used the mask (Figure 5) to evaluate the resulting connectivity. The values in Table 1 show that the method with the best performance is NURBS-T.

**Figure 10:**
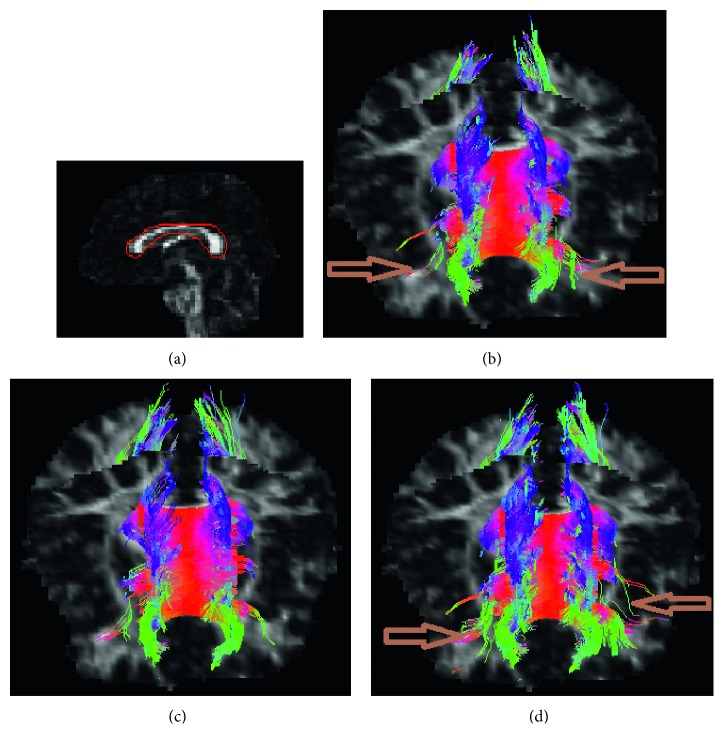
Fiber bundles tracked from ROI of corpus callosum. (a) ROI region, (b) multidirectional streamline, (c) NURBS-T, and (d) NURBS-G.

**Figure 11:**
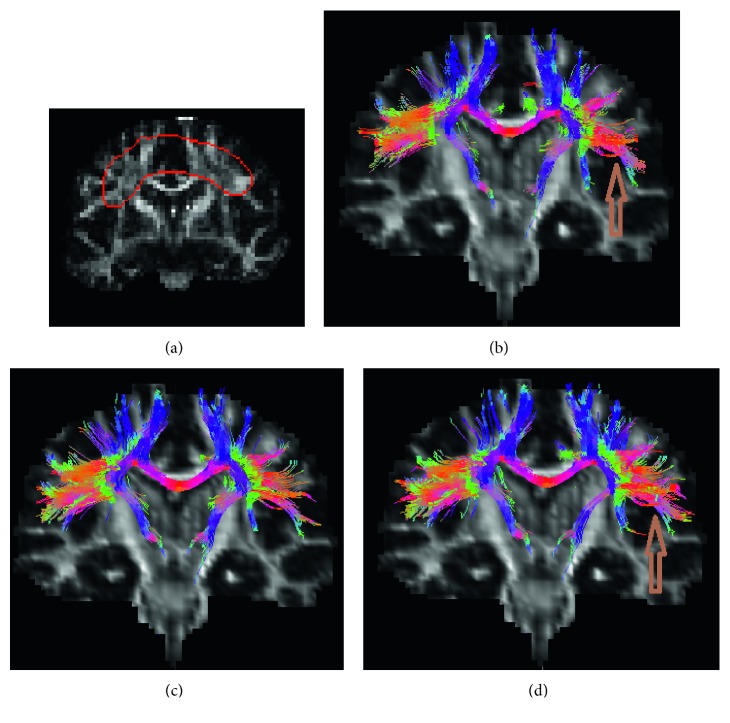
Fiber bundles generated from ROI of parietal lobe. (a) ROI region, (b) multidirectional streamline, (c) NURBS-T, and (d) NURBS-G.

**Figure 12:**
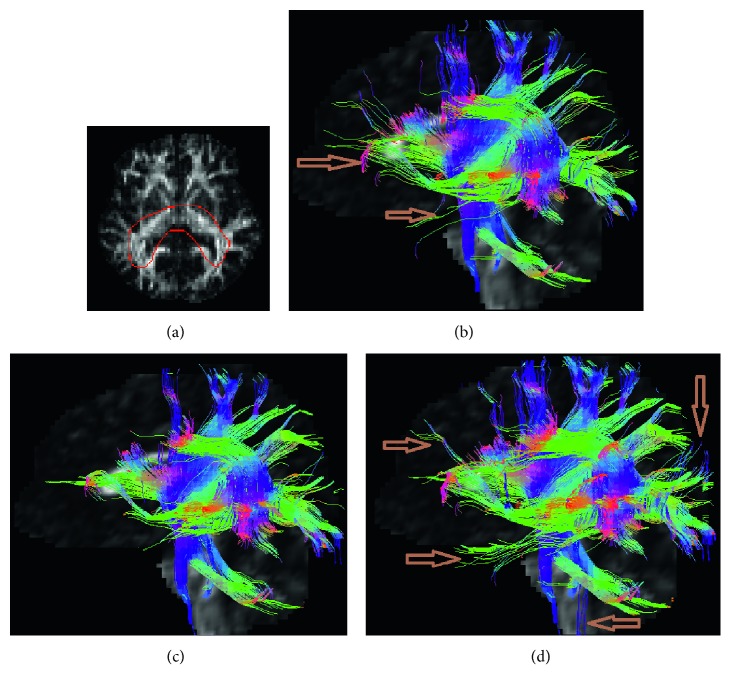
Fiber bundles tracked from ROI of bilateral mesial temporal lobes. (a) ROI region, (b) multidirectional streamline, (c) NURBS-T, and (d) NURBS-G.

Figures 10–12 show the estimated fibers of the in vivo human brain data. In this in vivo experiment, *θ*_th_ is 60° and *L*_th_ is 70 mm. *FA*_th_ is 0.15. We selected three ROIs to trace fiber pathways. The ROI in Figure 10 is located in the region of corpus callosum. The ROI in Figure 11 lies in the region of parietal lobe. The ROI in Figure 12 is in the region of bilateral mesial temporal lobes. As there is no golden standard of fiber distribution map with high resolution, we can only qualitatively analyze the results.

From Figure 10, we can easily pick out two fake fiber bundles that are marked by brown arrows. The thin bundle pointed by the left arrow is obviously nonexistent in the region of corpus callosum. The pathway pointed by the right arrow is unreasonable since it should not spread along the vertical direction. In Figure 10, from the morphological perspective, the fiber bundles are excessively messy and fluffy in the regions pointed by the two arrows because there are fewer constraints on the NURBS-G fitting. In Figures 11 and 11, there are too many crossing bundles, which disorderly emerge into the edge of WM in the region marked by arrows. In Figure 12, some unreasonable bundles could be found as their pathways spread out WM region. From Figure 12, we could see there are some minor bundles winds around the main bundles in the region pointed by the up-down arrow. In addition, the existence of the bundles in the regions pointed by the other three arrows is unreasonable.

From these in vivo tracking results, we can qualitatively validate our method. At last, to quantitatively analyze the proposed methods, we compared the results in the aspects of number of bundles, computation time, and storage (Table 2). The fiber bundles were stored as .mat file in Matlab 2014b. These methods were evaluated on the computer possessing 8G RAM and Intel Core i5-7200U CPU.

## 4. Discussion

In the presented study, we developed a novel tracking method based on NURBS curve fitting. The method consists of two steps. The first is to obtain the consecutive diffusion directions along a fiber pathway. The second is to carry out NURBS curve fitting. For the first step, we proposed a more effective way to find the consecutive vectors for a seed voxel among its 26-connected voxels. The comparison to FACT is shown in Figure 1. In the second step, the control points were obtained according to the equation given in the Algorithm 2. The corresponding weights are computed according to the equation given in the Algorithm 2. From the experimental results, we can conclude that the proposed method is well suited for exploring WM pathways.

The proposed method aims to reveal the connectivity among brain function areas. It is important to realize that our method does depend heavily on the parameters of control points and weights. Although we presented here both the theoretical foundation and a number of practical examples that characterize performance and accuracy of our approach, the main limitation of our work is the lack of a system wide analysis of the two parameters that can influence the fitting results. In NURBS fitting, we would continue to study the mathematical relationship between the weights and ODF peaks.

In general, there are two main factors influencing the tracking results: the noise in HARDI images and partial volume effects [40]. The noise could cause the inconsistency, and the incomplete information about partial volume effect could confuse the tacking process. In consequence, some fiber paths are incorrectly estimated [6]. Before the construction of ODF fields, we used NLPCA to denoise HARDI dataset. In the regions of fiber crossing, branching, and merging, the multiple compartments within a voxel make it hard to find out the fiber orientation from ODF fields for such entangled structures. In fact, the sensitivity to detect multiple fiber populations depends not only on the datasets but also on specifics of the construction technique of ODF. If the resolution capability of the construction method is low, the deviation between ODF maxima and the ground truth directions would become large. This error can limit the fiber tracking technique to fully delineate a fiber tract.

Another important factor that can influence the tracking results is stop criteria. FA could not be considered as one of the tracking stop criteria because FA is generally less than 0.2 in a voxel with crossing fibers [40]. Except for that, we considered the fiber length and the angle as stop criteria. However, validation of fiber tractography remains an open question [25].

## 5. Conclusion

Anatomical connectivity network is important to the investigation of human brain functions. The quality of anatomical connectivity relies on proper tract estimation [6]. In this work, we presented a novel algorithm based on NURBS curve fitting. The proposed methods exhibit promising potential in exploring the structural connectivity of human brain. They are easily implemented and proved efficient through phantom and real experiments. However, it is still difficult to identify the fiber bundles that are diverging, converging, and kissing. In future, our study will be mainly focused on how to solve this problem with NURBS fitting. More anatomical constraints should be used to guide tracking processes.

## Figures and Tables

**Algorithm 1:**
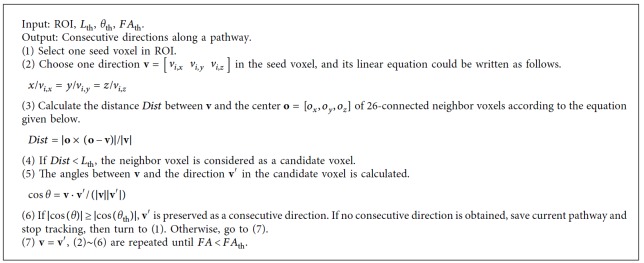
Summary of the method for extracting the consecutive directions along a pathway.

**Algorithm 2:**
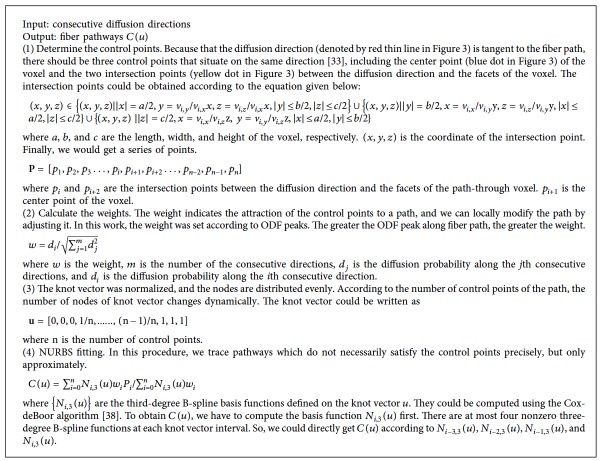
Summary of NURBS-T fiber tracking.

**Algorithm 3:**
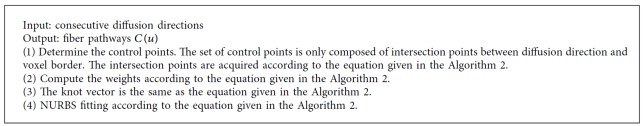
Summary of NURBS-G fiber tracking.

**Table 1:** The global connectivity evaluation of the fiber tracking algorithms.

	VC (%)	IC (%)	NC (%)	VB	IB
Streamline	73.7	3.1	23.2	15	10
NURBS-T	87.4	2.5	20.1	13	12
NURBS-G	79.7	5.9	21.4	16	13

**Table 2:** Comparison of in vivo tracking results.

ROI	Methods	Number of bundles	Computation time (second)	Memory storage (KB)
ROI1 (Figure 10)	Streamline	1065	85	9628
	NURBS-T	985	61	6677
	NURBS-G	1131	57	5246

ROI2 (Figure 11)	Streamline	811	66	10613
	NURBS-T	622	52	5646
	NURBS-G	703	49	4835

ROI3 (Figure 12)	Streamline	1570	102	11323
	NURBS-T	1526	71	7892
	NURBS-G	1703	59	6374

## Data Availability

The tractometer and real datasets used to support the findings of this study are available from the corresponding author upon request.
